# Mast Cell Neural Interactions in Health and Disease

**DOI:** 10.3389/fncel.2019.00110

**Published:** 2019-03-20

**Authors:** Aditya Mittal, Varun Sagi, Mihir Gupta, Kalpna Gupta

**Affiliations:** ^1^Vascular Biology Center, Division of Hematology, Oncology and Transplantation, Department of Medicine, University of Minnesota, Minneapolis, MN, United States; ^2^Department of Neurosurgery, University of California, San Diego, San Diego, CA, United States

**Keywords:** blood brain barrier, mast cell, endothelial cell, pain, inflammation, nervous system

## Abstract

Mast cells (MCs) are located in the periphery as well as the central nervous system (CNS). Known for sterile inflammation, MCs play a critical role in neuroinflammation, which is facilitated by their close proximity to nerve fibers in the periphery and meninges of the spinal cord and the brain. Multifaceted activation of MCs releasing neuropeptides, cytokines and other mediators has direct effects on the neural system as well as neurovascular interactions. Emerging studies have identified the release of extracellular traps, a phenomenon traditionally meant to ensnare invading pathogens, as a cause of MC-induced neural injury. In this review article, we will discuss mechanisms of MC interaction with the nervous system through degranulation, *de novo* synthesis, extracellular vesicles (EVs), tunneling nanotubes, and extracellular traps with implications across a variety of pathological conditions.

## Introduction

Mast cells (MCs) are proinflammatory cells that are the first responders of the immune system (Galli et al., 2005; Gupta and Harvima, 2018). MCs localize in proximity to afferent fibers innervating the periphery, visceral organs and meninges. MC proximity to the external environment makes them prime candidates for a rapid response against external stimuli and internal microenvironment. In addition to their function against the external environment, MCs are also sensitive to the endogenous environment and may thereby contribute to multiple pathobiologies, including pain, itch, and disorders of the nervous system (Mattila et al., 2011; Xanthos et al., 2011; Arac et al., 2014; Kempuraj et al., 2017; Gupta and Harvima, 2018). It is suggested that MCs contribute to pathology through interaction with the vasculature and the central nervous system (CNS). Upon activation, MCs quickly release substances from preformed granules including proteoglycans, proteases, leukotrienes, biogenic amines, and cytokines (Vukman et al., 2017). In addition to degranulation, MCs have a delayed response leading to the release of cytokines, neuropeptides, and chemokines by *de novo* synthesis. Noxious (toxic or injurious) substances released include but are not limited to histamine (Sjoerdsma et al., 1957), tryptase (Glenner and Cohen, 1960), chymase (Benditt and Arase, 1959), tumor necrosis factor α (TNFα; Gordon and Galli, 1990), and interleukin (IL-6), IL1β (Bradding et al., 1993; Nakamura et al., 2012), monocyte chemoattractant protein 1 (Vincent et al., 2013), nerve growth factor (Leon et al., 1994), brain-derived neurotrophic factor (Yuan et al., 2010), gonadotropin-releasing hormone (Khalil et al., 2003) and substance P (SP; Vincent et al., 2013; Taracanova et al., 2018). In this review article, we will examine the different ways in which MC activation interacts with the nervous system and their pathological implications.

## Localization of Mast Cells

MCs are specifically located in the dura mater/meninges of the spinal cord and brain (Khalil et al., 2007). Within the CNS, MCs are located on the abluminal side of the blood brain barrier (BBB) in apposition to astrocytes and neurons (Manning et al., 1994; Florenzano and Bentivoglio, 2000; Silverman et al., 2000). Increased BBB permeability can lead to MCs crossing into the CNS (Silverman et al., 2000). In addition, MCs can cross through the blood-spinal cord barrier (Dong et al., 2014). MCs are found in close proximity to sensory nerve endings, and their degranulation can modulate the excitability of nociceptors. In the spinal cord, white matter separates the dura from the lumbar dorsal horn allowing MC mediators to reach the superficial laminae, which is a key relay station that modulates synaptic transmission and nociception (Xanthos et al., 2011). It has been found that MCs can induce persistent nociception and long-term potentiation at spinal C-fiber synapses (Xanthos et al., 2011). This is mediated through SP release, which mediates pain sensation through unmyelinated C fibers (Tore and Tuncel, 2009). In most tissues, MCs and nerves have a gap of 20 nm allowing MCs to immediately act on peripheral nerves following degranulation. A correlation has been shown between MC proximity to nerve fibers and complex regional pain syndrome (Morellini et al., 2018). MCs also co-localize with astrocytes and may modulate the behavior of astrocytes to release more mediators through the release of histamine from MCs (Skaper and Facci, 2012).

## Mast Cell Activation and the Nervous System

### Mast Cell Degranulation

**Figure 1:**
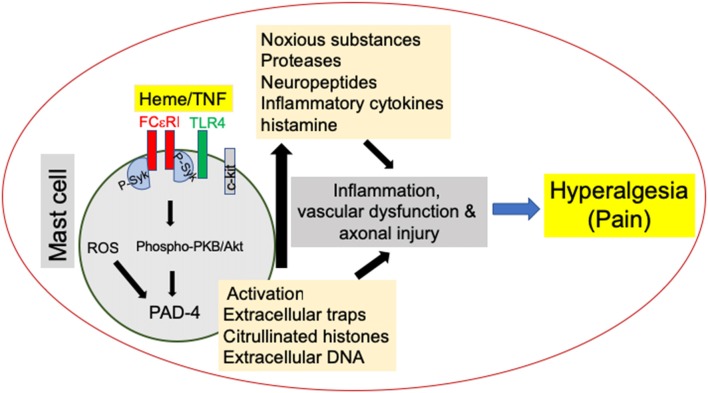
Mast cell (MC) activation promotes hyperalgesia in sickle cell disease (SCD). MC activation leads to the release of noxious substances such as proteases, neuropeptides and cytokines as well as the release of MC extracellular traps (MCETs) with citrullinated H3 histones and DNA, which cause noxious as well as physical injury to the vasculature and nerve bundles, leading to hyperalgesia in SCD. Emerging data suggest that this process is mediated by endoplasmic reticulum (ER) stress/reactive oxygen species (ROS) production and may engage FcεR1 and toll-like receptor 4 (TLR4) leading to Syk associated downstream protein kinase B (PKB/Akt) signaling. Consequent peptidylarginine deiminase-4 (PAD-4) activation may stimulate the release of extracellular traps, while inflammasome signaling may further augment inflammation.

Degranulation occurs within minutes of activation and results in the rapid release of substances from pre-formed granules. Degranulation begins with the activation of receptors with high affinity for IgE (FcεR1) and rearrangement of F-actin and microtubule formation. FcεR1 activates Fyn/Gab2/RhoA tyrosine kinases and leads to microtubule polymerization and the shuttling of secretory granules to the plasma membrane (Figure 1; Nishida et al., 2005). Tyrosine kinases Lyn and Syk of the activated FcεR1 cause calcium mobilization, vesicle fusion, and exocytosis of the granule through the protein kinase B (PBK)/Akt pathway and upregulation of peptidylarginine deiminase-4 (PAD-4) activation (Figure 1; Doyle et al., 2013; Aich et al., 2015). A variant of degranulation called transgranulation is associated with neuropathic pain (Keith et al., 1995; Wilhelm et al., 2005). Transgranulation occurs when MCs are in direct contact with other cells and thereby transfer the granules into nearby cells through exocytosis of the MCs and intake by the recipient cell. The effect is furthered through MC cytoplasmic extensions similar to pseudopodia that increase the range a MC can act through transgranulation (Barbara et al., 2004; Wilhelm et al., 2005). Because MCs are located closely to vasculature and nerve fibers, transgranulation can have a disruptive effect on those cells contributing to vascular dysfunction and neuropathic pain, respectively.

In the bladder, bidirectional communication between nerves and MCs using lamellipodia that enclosed fibers within a cell have been observed (Keith et al., 1995). Retrograde transport of MC mediators has also been shown in dorsal root ganglia (DRG; Murphy et al., 1999). In this study, a nerve injected with a MC degranulation product increased the expression of IL-6 mRNA in sensory neurons, and blockade of retrograde axonal transport attenuated the induction of IL-6 mRNA in primary sensory neurons.

### *De novo* Synthesis

As a late response, MCs release cytokines and chemokines that are synthesized *de novo*. The mechanism for activation is thus complex and involves multiple receptors. Immunoglobulin E (IgE) receptors are involved in mediating response in cooperation with the toll-like receptor (TLR). TLRs also recruit CD14 or CD48 for the effect of TLR ligands (Marshall, 2004).

IgE independent receptors also regulate MC *de novo* synthesis. This involves the receptor tyrosine kinase kit (*c-kit)*, which is a receptor tyrosine kinase for stem cell factor (SCF; Mitsui et al., 1993). SCF promotes the development of more MCs. The large number of cytokines, chemokines, and neuropeptides released from MCs along with their proximity to axonal processes may be a large contributor to neuropathy and inflammation.

### Extracellular Vesicles

Extracellular vesicle (EV) release is common to many different cells. EVs may be formed in endosomes and released through exocytosis. MC exosomes are known to interact with sensory nerves through the release of SP (Azimi et al., 2017). In turn, SP interacts with Mas-related G protein-coupled receptors (Mrgprs) to activate DRG neurons in mice (Azimi et al., 2017). Neurokinin 1 (NK1) receptors and Mrgprs were activated in mice and then pain behaviors were measured *in vivo* and DRG activation was measured in culture leading to the conclusion that SP activates DRG in culture through Mrgprs not NK-1 receptors (Azimi et al., 2017).

Because of their small size, EVs can travel long distances and influence synaptic transmission in the CNS. EVs may also participate in reuptake by local neurons as EVs can be localized as MC granules (Groot Kormelink et al., 2016). Groot Kormelink et al. (2016) found that upon activation of MCs a CD63-positive subset of EVs is released. Upon phospholipid and proteome analysis of these EVs, it was found that the EVs contain MC-specific mediators (Groot Kormelink et al., 2016).

MC-released EVs can influence dendritic cell maturation *via* immunomodulatory exogenously encountered antigens (Ags; Skokos et al., 2003). Ags induce phenotypic changes in dendritic cell maturation by up-regulating MHC class II, CD80, CD86, and CD40 molecules. These molecules stimulate T lymphocytes and induce Ag-specific immune responses which could impact the function of dendritic cells.

MicroRNAs contained in EVs may also participate in neuropathy. MiRNA-let-7b causes rapid excitation in DRG neurons *via* TLR7 and transient receptor potential ankyrin 1 (TRPA1; Park et al., 2014). MiRNA-let-7b causes rapid inward currents and exciting the DRG neurons and inducing pain *via* TLR7 and TRPA1 (Park et al., 2014).

### Tunneling Nanotubules

MC form tunneling nanotubules (TNTs), which are F-actin structures that form in response to reticulation. TNTs are similar to pseudopodia described earlier, except they are not adherent, and can span distances longer than pseudopodia. MC-microglia interactions have been found to be involved in brain inflammation (Skaper and Facci, 2012). MCs cultured in medium containing MC activators were found to rapidly form TNTs to transport mitochondrial and secretory granule particles to other MCs and glioblastoma, implicating TNTs in MC-microglia interactions (Weng et al., 2016).

### Mast Cell Extracellular Traps

Extracellular trap formation is a process to ensnare external organisms for self-defense. This process involves citrullination of histone proteins, resulting in disassembly of DNA and ejection of web-like contents (Jorch and Kubes, 2017).

MC extracellular trap (MCET) formation is dependent on reactive oxygen species (ROS) formation and engagement of TLR4 (Figure 1; Stoiber et al., 2015). In addition to DNA, MCETs also contain fibers with tryptase. MCETs have been suggested to contain chemokines and cytokines necessary for an inflammatory response, which could lead to tissue damage, inflammation, and neuronal activation (Schauer et al., 2014; Möllerherm et al., 2016). IL-17 and IL-8 have been shown to be released by MCETs. MCs are the majority of IL-17-containing cells in control and psoriatic skin (Lin et al., 2011). Interestingly, MCETs showed bright costaining for IL-17. MCs and neutrophils have been reported to release IL-17, which contributes to the pathology of psoriasis.

## Neurovascular Interactions and Pathological Outcomes

It is well documented that MC mediators contribute to endothelial dysfunction in the vasculature (Kunder et al., 2011). Excess of MC mediators can cause an increase in BBB permeability. It is known that activation of MCs locally increases BBB permeability (Zhuang et al., 1996). Zhuang et al. (1996) found that upon MC degranulation by C48/80 treatment, BBB permeability was increased.

MCs operate in a feed-forward mechanism. Inflammation caused by MCs can further activate the MCs in an autocrine manner. Mediators released by MCs such as IL33 and tumor necrosis factor alpha (TNFα) have been known to activate MCs (Taracanova et al., 2017). SP along with IL33 causes MCs to increase secretion and gene expression of IL-1β (Taracanova et al., 2018). These responses were mediated by SP and IL-33 receptors, NK1 and small conductance Ca^2+^-activated K^+^ (SK2), respectively on MCs. Receptors were inhibited by methoxyluteolin inhibiting IL33 decreased IL-1β release (Taracanova et al., 2017).

MCs have been found to increase vascular permeability in the skin of mice with sickle cell disease (SCD; Vincent et al., 2013). Treatment of mice with a MC inhibitor cromolyn or imatinib or cannabinoids reduced vascular permeability in these mice (Vincent et al., 2016). These observations led to the suggestion that MC activation leads to the release of SP which activates protease-activated receptor 2 on the peripheral nerve endings, which in turn release more neuropeptides including SP leading to vascular dilatation and increased permeability. Thus MC activation leads to neurogenic inflammation involving neurovascular interactions.

Increased vascular permeability has been found in the CNS where MC degranulation compromises the BBB and allows further entry of inflammatory substances into the brain (Zhuang et al., 1996). Acute stress has pro-inflammatory effects that are mediated through the activation of MCs *via* corticotropin-releasing hormone (Esposito et al., 2001). Additionally, external and internal ROS formation contributes to changes in endothelial cell-cell interactions (van Wetering et al., 2002), BBB integrity (Lehner et al., 2011), and the disruption of tight junctions (Schreibelt et al., 2007). Increase in BBB permeability is associated with higher levels of neuroinflammation and brain dysfunction. Additionally, BBB disruption may further changes by systemic inflammation (Dénes et al., 2011; Knowland et al., 2014). Dénes et al. (2011) found that systemic inflammation compromises survivability after stroke which also augments BBB damage. Activation of meningeal MCs has been shown to worsen stroke pathology in mice (Arac et al., 2014). Therefore, activation of MCs in the periphery, as well as CNS, has implications in altering the neural activity and function directly and/or *via* neurovascular interactions.

## Conclusion

MCs contribute to neural and vascular injury directly as well as induce neurovascular interactions. The complex milieu of multiple mediators released from MCs *via* diverse mechanisms alters the microenvironment leading to a hypersensitized system. Recent advances in MC-CNS interactions demonstrate that MC-induced hypersensitivity also contributes to the CNS disorders and pain. Thus, targeting of MCs provides a potentially treatable target for the disorders of the CNS and pain.

## Author Contributions

AM wrote the manuscript and prepared for submission. VS edited the manuscript. MG wrote and edited the manuscript. KG conceived, designed, and supervised the manuscript writing and editing and prepared the figure.

## Conflict of Interest Statement

KG is a Consultant for Tau Tona Group and Novartis but it does not have conflict with the present work. The remaining authors declare that the research was conducted in the absence of any commercial or financial relationships that could be construed as a potential conflict of interest.

## Abbreviations

Ags: exogenously encountered antigens
BBB: blood brain barrier
c-kit: receptor tyrosine kinase kit
CNS: central nervous system
DRG: dorsal root ganglion
EV: extracellular vesicle
FcεR1: receptors with high affinity for IgE
IgE: immunoglobulin E
IL: interleukin
MC: mast cell
MCET: mast cell extracellular trap
MS: multiple sclerosis
NK1: neurokinin 1
PAD: peptidylarginine deiminase
PBK/Akt: protein kinase B
ROS: reactive oxygen species
SCF: stem cell factor
SCD: sickle cell disease
SK2: small conductance Ca^2+^-activated K^+^
TLR: toll-like receptor
TNFα: tumor necrosis factor α
TNT: tunneling nanotubules
TRPA1: transient receptor potential ankyrin 1.
